# Prevalence and Risk Factors of Violence by Psychiatric Acute Inpatients: A Systematic Review and Meta-Analysis

**DOI:** 10.1371/journal.pone.0128536

**Published:** 2015-06-10

**Authors:** Laura Iozzino, Clarissa Ferrari, Matthew Large, Olav Nielssen, Giovanni de Girolamo

**Affiliations:** 1 Psychiatric Epidemiology and Evaluation Unit, IRCCS Centro San Giovanni di Dio Fatebenefratelli, Brescia, Italy; 2 Department of Public Health and Community Medicine, Section of Psychiatry, University of Verona, Verona, Italy; 3 Prince of Wales Hospital Sydney, Sydney, Australia; 4 St. Vincent’s Hospital Sydney, Sydney, Australia; 5 University of New South Wales, Sydney, Australia; Peking University, CHINA

## Abstract

**Background:**

Violence in acute psychiatric wards affects the safety of other patients and the effectiveness of treatment. However, there is a wide variation in reported rates of violence in acute psychiatric wards.

**Objectives:**

To use meta-analysis to estimate the pooled rate of violence in published studies, and examine the characteristics of the participants, and aspects of the studies themselves that might explain the variation in the reported rates of violence (moderators).

**Method:**

Systematic meta-analysis of studies published between January 1995 and December 2014, which reported rates of violence in acute psychiatric wards of general or psychiatric hospitals in high-income countries.

**Results:**

Of the 23,972 inpatients described in 35 studies, the pooled proportion of patients who committed at least one act of violence was 17% (95% confidence interval (CI) 14–20%). Studies with higher proportions of male patients, involuntary patients, patients with schizophrenia and patients with alcohol use disorder reported higher rates of inpatient violence.

**Conclusion:**

The findings of this study suggest that almost 1 in 5 patients admitted to acute psychiatric units may commit an act of violence. Factors associated with levels of violence in psychiatric units are similar to factors that are associated with violence among individual patients (male gender, diagnosis of schizophrenia, substance use and lifetime history of violence).

## Introduction

Physical violence in acute psychiatric wards can be a major problem [1, 2], not only because of the potential for injury to patients and staff, but also because of the counter therapeutic effects of both violence and measures to prevent violence. The emotional effects of exposure to physical violence on other inpatients can include anger, shock, fear, depression, anxiety and sleep disturbance [3]. Staff surveys show that between 75% and 100% of nursing staff on acute psychiatric units have been assaulted by a patient at some stage in their careers [4, 5]. Physical violence against staff is thought to contribute to low morale, high rates of sick leave and high staff turnover, [6, 7] which can trigger a vicious cycle, as low staffing levels and the presence of temporary staff can lead to more adverse incidents [8]. The consequent reliance on temporary staff increases service costs and has been linked to lower standards of care [9]. Moreover, the perceived threat of violence may result in greater use of coercive measures such as seclusion, restraint and enforced medication, which patients often describe as traumatic [10] and can, in turn, trigger aggressive responses from patients instead of engagement and cooperation with treatment [11, 12].

There is a wide variation in the reported rates of violence in acute inpatient settings, which might be due to real differences in the rates of violence between wards, differences in the definition of violence, differences in the duration of measurement and methods of data collection, and variations in the level of under-reporting of aggressive incidents by mental health care workers [13]. Several recent reviews have examined the socio-demographic and clinical variables associated with inpatient aggression and violence in individual patients. Cornaggia and colleagues [14] performed a narrative review of factors associated with inpatient violence, and concluded that a history of previous aggressive incidents, longer hospitalization, involuntary admission, impulsiveness, hostility, and the aggressor and victim being of the same gender were the most important factors associated with acts of inpatient violence. More recently Dack and colleagues [15] performed a meta-analysis of studies of factors associated with either aggression or violence in a diverse range of inpatient settings including acute psychiatric wards, psychiatric intensive care units and forensic wards. They found aggression to be associated with young age, male sex, involuntary admission, not being married, a diagnosis of schizophrenia, a greater number of previous admissions, a history of violence, a history of self-destructive behaviour and a history of substance use. Hence the factors associated with inpatient violence appear to be similar to those associated with violence among outpatients and in the wider community. It would not be surprising to find that wards that admit patients who are more likely to be violent have higher rates of violent incidents. However, the factors associated with overall rates of violence in acute psychiatric wards are not known.

A better understanding of the factors associated with violence in acute psychiatric wards would assist in the planning of services, the development of preventative measures and in comparing the performance of services. Moreover, knowing the factors influencing the rates of violence at a ward level might assist in interpreting reported rates of violence in particular wards.

The aims of this study were to use systematic meta-analysis in order to estimate the pooled rate of violence, in terms of period prevalence, in acute psychiatric wards, and to explore the aggregate level ward characteristics that might explain the variation in the reported rates of violence between wards.

## Methods

The methods conformed to the Preferred Reporting Items for Systematic Reviews and Meta-Analyses (PRISMA) guidelines [16].

### Search Strategy

Studies were identified by searching the electronic databases Pubmed, Scopus and Cumulative Index to Nursing and Allied Health Literature (CINAHL). Queries were limited to articles published between January 1995 and December 2014 and reporting data on violence in adult psychiatric inpatients, using the following search terms: (“violence” OR “aggression” OR “aggressive behavior” OR “assault”) AND (“mental disorders” OR “psychosis” OR “acute psychiatric inpatients”) AND (“hospital” OR “hospitalization” OR “acute psychiatric wards”) in either the title or the abstract. Where applicable, age (19+ years, 19–24 years, 19–44 years, 45–64 years) and publication date (1995–2014) filters have been used (exact search sequence with filters are specified in S1 Fig). The reference lists of the articles identified by the electronic searchers were hand searched for further relevant studies. Only articles published in peer-reviewed journals were considered, in order to limit the searches to studies with an adequate level of methodological rigor.

We chose 1995 as the starting point for the searches, in order to examine a 20 years period, and in recognition of the different way hospital care was provided, and differences in the way adverse events might have been recorded in previous decades.

### Inclusion and exclusion criteria

We included studies that reported the proportion of adult patients admitted to acute psychiatric wards in high-income countries (http://www.worldbank.org/) who had committed at least one act of violence during hospitalization. Violence was defined as any incident in which a patient harmed or attempted to physically harm another person, including fellow patients, hospital staff or a visitor to the ward. Studies that reported only rates of verbal hostility and self-harm behaviour were excluded; as well as studies that reported the proportion of violent patients for which it was not possible to discriminate among different types of aggressive behaviours. In addition, we excluded articles which contained the proportion of “aggressive patients” and the number of physical violence episodes, but in which it was not possible to obtain the number of patients who had committed physical violence. We included studies on violence that occurred in General Hospitals Psychiatric Wards (GHPWs) and Acute Psychiatric Wards of stand alone psychiatric hospitals (APWs). Studies conducted in forensic hospitals and the forensic wards of other psychiatric hospitals were excluded, as were studies performed in outpatient settings, on patients hospitalized in non-psychiatric emergency wards, in long stay wards that did not accept acute admissions and in any type of non-hospital residential facility. We included studies of violence committed by adult patients; studies conducted in wards admitting only adolescents (up to 18 years of age) or psycho-geriatric patients (older than 65) were excluded. Finally, we included studies from 31 countries classified as high-income countries by the World Bank, but did not include studies from low and middle-income countries for two reasons: the characteristics of inpatient psychiatric wards (and also the systems of mental health care) are often profoundly different in middle and low-income countries, and a comparison of rates of violence in very different settings would be inappropriate. In addition a 2002 World Bank study [17] found out that violence rates in the general population and inequality are positively correlated within countries and also between countries; therefore it seemed inappropriate to compare violence in hospital settings between countries with very different rates of violence in the general population.

Rate of violence was expressed in term of period prevalence, as we considered only patients who had committed at least one act of violence during hospitalization (regardless of any previous aggressive behaviors).

### Study selection and data extraction

The searches yielded a total of 16,509 titles and abstracts that were screened for potential relevance, from which 10,170 articles were selected for further consideration after the removal of duplicate publications. The abstracts of these 10,170 papers were reviewed, leaving 531 potentially relevant studies that were examined in more detail. Of these, 35 met inclusion criteria. (S1 Fig).

Data extracted were: year of publication, country in which the study was conducted, setting, average of beds in the ward, average length of stay, sample size, mean age of patients, number of patients who committed an act of violence, number of males, number of admissions, whether the patient was admitted involuntarily, psychiatric diagnoses, lifetime history of alcohol and drug abuse, history of violence and the method for recording acts of violence (Staff Observation Aggression Scale,–SOAS-, the Staff Observation Aggression Scale-Revised,-SOAS-R-, the Overt Aggression Scale,–OAS-, the Modified Overt Aggression Scale,–MOAS-, or others). Satisfactory intercoder reliability and agreeability were established for the data extraction (97%, range: 85%–100%; *k* = 35) and screening (95.6%, range: 85%–100%; *k* = 35) respectively. The studies included in the review and in the meta-analysis are reported in Tables 1 and 2.

**Table 1:** Summary information of the studies included in meta-analysis.

Author, Year	Country	Multiple (M) or Single (S) study	Average number of beds	Sample (N)	Number of violent patients	Male gender (N)	Average length of stay (days)	Admissions (N)	Involuntary admissions (N)	Mean age
Aberhalden et al, 2006 [44]	Switzerland	M	4	519	37	315	22.4	519	319	37.8
Aberhalden et al, 2008 [45]	Switzerland	M	-	2.364	314	1.262	19.0	2.364	1.040	39.5
Amore et al, 2008 [38]	Italy	S	20	303	75	182	-	374	-	41.6
Ash et al, 2003 [46]	Australia	S	20	119	26	83	-	143	51	35.0
Barlow et al, 2000 [37]	Australia	M	16	1.269	174	662	13.8	2.536	-	37.0
Beauford et al, 1997 [47]	USA	S	-	328	38	170	16.0	328	285	41.9
Biancosino et al, 2009 [30]	Italy	M	-	1.324	37	677	-	1.324	178	-
Bjorkdahl et al, 2006 [48]	Sweden	S	10	73	11	37	13.6	73	-	39.6
Boggild et al, 2004 [39]	USA	S	36	105	44	-	-	259	-	40.5
Bowers et al, 2003 [49]	UK	M	-	238	38	140	-	238	100	40.0
Carr et al, 2008 [50]	Australia	M	21	3.877	551	2.210	14.6	5.546	2.640	37.1
Cohen et al, 2008 [51]	Ireland	S	18	99	18	50	-	99	-	40.0
Cookson et al, 2012 [52]	Australia	M	25	79	35	43	19.6	310	212	40.8
Daffern et al, 2010 [53]	Australia	S	50	122	10	-	15.0	395	63	39.5
Dumais et al., 2012 [54]	Canada	S	-	77	16	47	-	77	-	36.4
Eaton et al, 2000 [55]	UK	S	20	52	17	46	-	79	-	32.0
Ehmann et al., 2001 [56]	Canada	S	20	78	20	53	-	78	-	34.5
Grassi et al, 2001 [57]	Italy	S	15	1.534	116	798	12.8	2.461	674	39.5
Hartvig et al, 2011 [58]	Norway	M	41	1.017	92	536	15.0	1.469	389	42.7
Ketelsen et al, 2007 [59]	Germany	M	17	2.210	171	1.226	28.2	2.246	323	46.9
Krakowski&Czobor, 2004 [60]	USA	M	-	1.487	222	1.026	-	1.487	-	36.1
Lam et al, 2000 [61]	USA	S	-	390	76	205	-	390	344	41.8
Mauri et al, 2011 [62]	Italy	S	15	244	82^c^	179	12.2	244	74	41.9
Mellesdal, 2003 [63]	Norway	S	12	934	98	476	11.6	1.507	1.053	41.1
Nijman et al, 1997 [64]	Netherlands	S	20	123	31	-	-	123	-	-
Nijman et al, 2002 [65]	Netherlands	S	20	89	23	54	-	98	38	36.0
Oulis et al, 1996 [66]	Greece	M	-	136	8	72	-	136	-	-
Owen et al, 1998 [67]	Australia	M	-	855	174	-	-	855	427	-
Raja et al, 1997 [68]	Italy	S	12	313	22	143	-	360	36	41.8
Raja et al, 2005 [69]	Italy	S	12	2.395	70	1.067	-	2.395	604	41.9
Ross et al, 2012 [70]	UK	M	-	522	110	279	-	522	-	41.0
Saverimuttu& Lowe, 2000 [71]	UK	S	5	170	57	136	-	170	-	-
Soliman&Reza, 2001 [40]	UK	M	15	329	49	239	36.4	474	50	39.6
Troisi et al, 2003 [72]	Italy	S	-	80	20	80	-	80	24	34.1
Valeer et al, 2011 [73]	Norway	M	4	118	13	66	5.6	118	57	36.3

^c^Information was obtained from the author.

**Table 2:** Additional information of the studies included in meta-analysis.

Author, Year	Diagnosis of schizophrenia	Diagnosis of bipolar disorder	Diagnosis of personality disorder	Previous history of violence	Lifetime history of alcohol or/and drug abuse	Measurements	Quality score (range 0–4)
Aberhalden et al, 2006 [44]	196	-	-	-	120	SOAS-R, BVC	4
Aberhalden et al, 2008 [45]	734	-	-	-	574	SOAS-R, BVC	4
Amore et al, 2008 [38]	151	26	85	105	117	OAS	4
Ash et al, 2003 [46]	56	-	49	-	57	Notspecified	3
Barlow et al, 2000 [37]	271	91	38	-	-	AIF	4
Beauford et al, 1997 [47]	75	71	-	-	-	OAS	3
Biancosino et al, 2009 [30]	472	206	168	-	117	Retrospective chart records	4
Bjorkdahl et al, 2006 [48]	15	14	9	-	-	SOAS-R	3
Boggild et al, 2004 [39]	0	-	-	30	76	nursing chart record	3
Bowers et al, 2003 [49]	169	-	-	-	-	PCC	3
Carr et al, 2008 [50]	1,473	543	733	318	1,729	AIF, OAS	4
Cohen et al, 2008 [51]	26	23	9	-	8	SOAS-R	3
Cookson et al, 2012 [52]	-	-	-	-	-	OAS	3
Daffern et al, 2010 [53]	-	-	-	-	-	OAS	3
Dumais et al., 2012 [54]	40	26	5	-	-	DASA	3
Eaton et al, 2000 [55]	33	-	-	40	39	Clinical record and hospital incident forms	1
Ehmann et al., 2001 [56]	53	9	-	35	28	MOAS	4
Grassi et al, 2001 [57]	632	136	216	-	31	SOAS	4
Hartvig et al, 2011 [58]	208	122	92	-	-	REFA	3
Ketelsen et al, 2007 [59]	512	236	102	-	924	SOAS	4
Krakowski&Czobor, 2004 [60]	-	-	-	-	-	MOAS	3
Lam et al, 2000 [61]	87	87	-	53	102	Retrospective records	2
Mauri et al, 2011 [62]	130	17	51	-	147	MOAS	4
Mellesdal, 2003 [63]	290	523	141	-	157	REFA	4
Nijman et al, 1997 [64]	-	-	-	-	-	SOAS	3
Nijman et al, 2002 [65]	50	-	-	-	-	SOAS-R	3
Oulis et al, 1996 [66]	88	11	-	-	-	MOAS	4
Owen et al, 1998 [67]	-	-	-	-	-	Violence and AggressionChecklist	4
Raja et al, 1997 [68]	110	82	32	-	34	nursing chart record	4
Raja et al, 2005 [69]	295	386	55	-	437	Modified version of the Morrison's scale	4
Ross et al, 2012 [70]	215	-	-	-	-	PCC	3
Saverimuttu& Lowe, 2000 [71]	73	34	18	-	21	Violent incident forms	2
Soliman&Reza, 2001 [40]	110	53	44	52	122	SOAS	3
Troisi et al, 2003 [72]	53	20	3	-	4	MOAS	4
Valeer et al, 2011 [73]	54	-	-	-	33	SOAS-R	4

### Assessment of study quality

Study quality was assessed using a four-point “strength of reporting” scale, derived from the Strengthening the Reporting of Observational Studies in Epidemiology (STROBE) statement checklist [18]. As observed by Sanderson and colleagues [19], there is no single tool that is suitable for assessing the quality of all types of observational studies. However, as the STROBE provided a starting point for the development of quality assessment tools, and is the most widely used method of assessing study quality, we decided to use STROBE items to assess the quality of our primary studies.

A score of 1 was accorded if each of the following methodological features were present: (i) recruitment by consecutive patients, (ii) data collected prospectively, (iii) the presence of detailed definitions of outcomes, exposures, predictors, potential confounders, and effect modifiers; and (iv) data and details of methods of violence assessment: structured and semi-structured measurement methods. The sample of included studies was dichotomized according to strength of reporting score of <3 or ≥3 in order to assess the association between study quality and the reported proportion of violent patients.

### Data Analysis

Random effects meta-analysis was used to calculate a pooled estimate of the proportion of inpatients who committed an act of violence and 95% confidence intervals (CI). A random-effects model was chosen as conservative approach because we assumed that there would be significant between study heterogeneity in patient populations and methods of measurement of violence. The *Q* statistic and I^2^ index (% of total variability due to heterogeneity) were used to assess heterogeneity among studies. A significant Q value, and high (larger than 50) I^2^ index indicate lack of homogeneity of findings among studies [20].

Outlying studies were detected using externally standardized residual (SR) values, DFFIT values and Cook’s distances (CD) [21].

Several characteristics were identified to analyse their effect on violence prevalence. Categorical characteristics were treated as moderators and effectiveness was compared across subgroups formed by country (with categories: Europe, UK-Ireland, US-Canada, Australia), year of publication (1995–2004 vs 2005–2014); violence measurement tool (SOAS/OAS/MOAS vs other tools); study setting (single vs multicenter) and study quality (less than 3, or greater than or equal to 3). The study size effect was evaluated through the computation of power per study as defined by Turner and colleagues [22] (i.e. defining an adequate power as ≥50% power to detect relative risk reduction of 30%). The new categorical variable, study power (underpowered vs powered), was incorporated as moderator. Continuous characteristics including number of beds in the ward, mean age, average length of stay in days, proportion on the study sample of: males, total number of admissions, total number of involuntary admissions, patients with bipolar disorder, patients with schizophrenia, patients with personality disorders, patients with alcohol use disorder and patients with lifetime history of violence, were examined as covariates using random effects (restricted maximum-likelihood estimation) meta-regression. Finally, multivariable meta-regression analyses were carried out to detect any unexplained between study heterogeneity.

Publication bias was evaluated by rank correlation test (Begg’s test) [23] and Duval and Tweedie’s “Trim and fill” method [24]. With regard to the second method, the right most studies considered symmetrically unmatched are trimmed. The trimmed studies are then replaced and their missing counterparts imputed or “filled”. This then allows for the computation of an adjusted effect size and confidence interval [25].

All statistical analyses were performed using R: A language and environment for statistical computing, version 3.03 (R Core Team, 2013; R foundation for Statistical Computing) and its R-metafor package [26, 27]. The level of statistical significance was set at p < 0.05.

## Results

**Fig 1:**
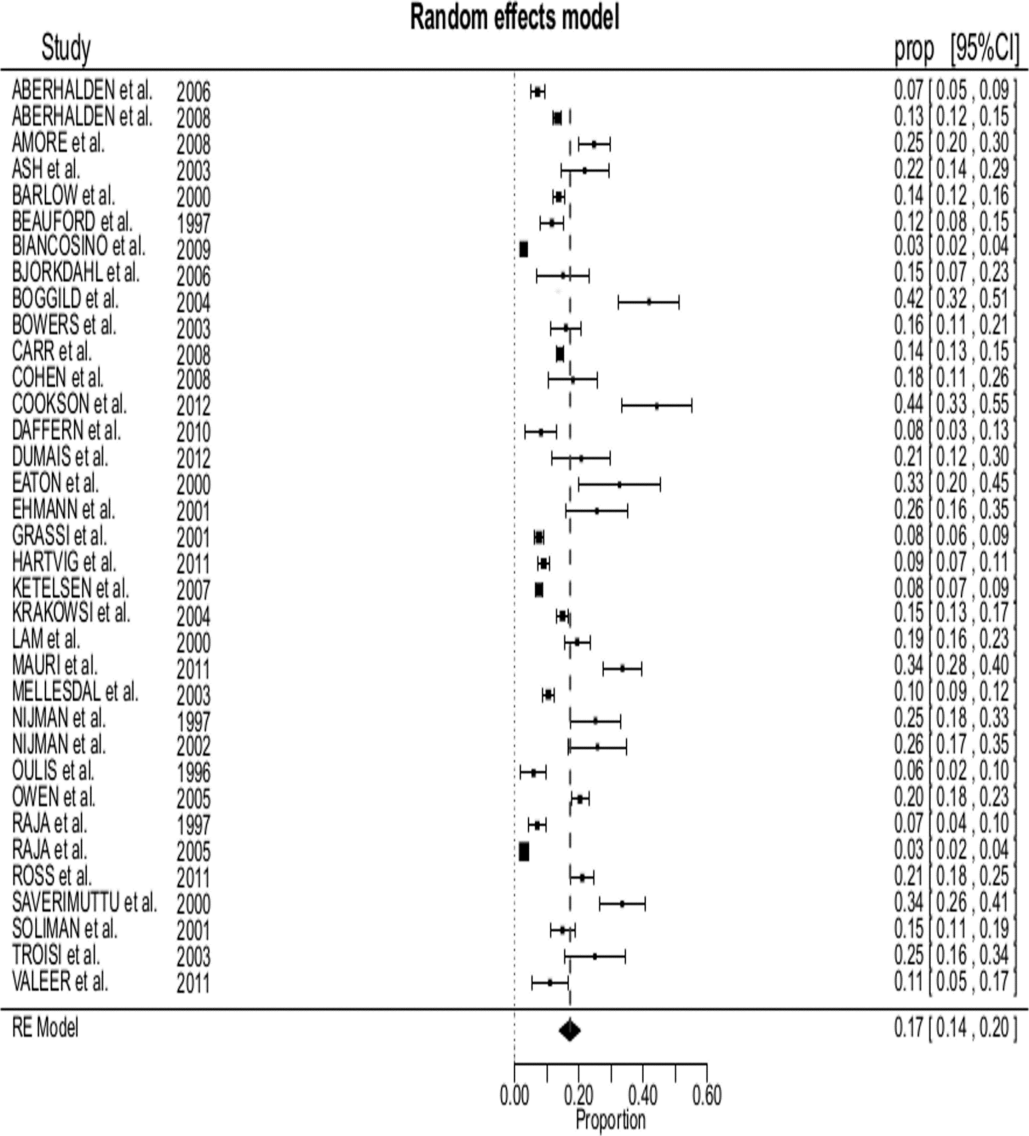
Forest plot of all 35 selected studies: prevalence of violence estimates (boxes) with 95% confidence limit (bars); pooled prevalence is reported as diamond.

The 35 included studies reported on 23,972 patients. Tables 1, 2 and Fig 1 show detailed information from the 35 selected studies.

Twenty-three out of 35 studies were from Europe (Italy 7, United Kingdom and Ireland 6, Norway 3, The Netherlands and Switzerland 2, and Germany, Sweden and Greece 1). There were 6 studies from Australia, 4 from the United States and 2 studies from Canada.

In every study, data on violent incidents were collected using standardized incident forms. More than half of the studies (N = 20) used SOAS, SOAS-R, OAS and MOAS. These scales have a range of definitions of violence: for example, in the SOAS, an aggressive patient is defined as being reported by staff as having an incident of just physical aggression against others, whereas the MOAS includes incidents of verbal aggression, physical violence against objects and violence against self, as well as physical violence against others. However, in each of the studies that used the MOAS we were able to extract the number of patients who committed an act of physical violence against another person.

### Meta-analysis of possible factors associated with in-patient violence

The pooled prevalence of inpatients who committed at least one act of violence was 17% (95%CI 14–20%, range 3% to 44%) in a heterogeneous set of studies, *Q*(34) = 1185.7, *p* = < .001 (see Fig 1).

None of the influential case measures reported extreme values so no outliers were detected.

The proportion of violent patients was significantly greater in subgroups of studies rated as being of lower study quality (28%, 95%CI [18–37] vs 16% 95%CI[13–20]) (Table 3). We performed a sensitivity analysis to check the extent to which the presence of lower quality studies influenced the total effect size, an estimated pooled prevalence. Combined effect sizes and confidence boundaries were recomputed after the three lower quality studies were removed. The results (16% 95%CI[13–20]) showed that the pooled proportion did not significantly change. No significant differences in pooled prevalence were detected in the other subgroup analysis.

**Table 3:** Subgroup analysis for prevalence of violence.

Study subgroups	N° of studies	N° of studies		Heterogeneity				Publication bias	
				I^2^(%) *	Group heterogeneity			Begg’s test	
					Q	df(Q)	p	Tau	p-value
*Total*	*35*	*17*	*[14 – 20]*	*98.7*	*1185.7*	*33*	*<0.001*	*0.1*	*0.179*
**Country group**									
Europe	17	13	[9 – 18]	99.0	492.5	16	<0.0001	0.3	0.177
UK-Ireland	6	22	[16 – 28]	87.0	27.7	5	<0.0001	0.3	0.469
US-Canada	6	22	[13–30	95.2	44.4	5	<0.0001	0.5	0.272
Australia	6	19	[11 – 28]	98.1	57.0	5	<0.0001	0.5	0.272
**Year of the study**									
(1995–2004)	18	19	[14 – 23]	96.9	224.6	17	<0.0001	0.4	**0.021**
(2005–2014)	17	16	[11 – 20]	99.2	830.5	16	<0.0001	0.1	0.439
**Quality score**									
<3	3	28	[18 – 37]	81.9	13.7	2	0.001	0.3	1.000
≥3	32	16	[13 – 20]	98.7	1091.8	31	<0.0001	0.2	0.109
**Measurement tool**									
MOAS-SOAS-OAS	15	18	[12 – 23]	99.0	616.2	14	<0.0001	0.1	0.559
Other tools	20	17	[13 – 21]	98.1	306.9	19	<0.0001	0.3	0.074
**Study Setting**									
Single	20	20	[15 – 25]	97.7	546.5	19	<0.0001	0.2	0.233
multicenter	15	14	[10 – 18]	98.5	546.8	14	<0.0001	0.1	0.697
**Study power**									
Underpowered	12	19	[12 – 27]	97.6	238.4	11	<0.0001	0.4	0.063
powered	23	16	[13 – 20]	98.5	871.8	22	<0.0001	0.1	0.373

* All p-values of I^2^ are <0.001.

The univariate meta-regression models revealed that the proportion of male patients explained more than 40% of between study heterogeneity. A similar percentage of heterogeneity was explained by lifetime history of violence (39.7%). Other characteristics of the primary research that explained between study heterogeneity were the proportion of patients with alcohol abuse disorders (35.3%), the proportion who had an involuntary admission (26.5%) and the proportion of patients with a diagnosis of schizophrenia (20.5%). In all univariate models, the residual heterogeneity was high (Table 4).

**Table 4:** Meta regression results: univariate and multivariable models.

	Risk factor	Type of data	Coefficient	Coefficient p-value	Explained heterogeneity R^2^ (%)	Test for residual heterogeneity			Begg’s test	
						Q_E_	df	p-value	Tau	p-value
**Univariate models**	**Ward size**	**(average N. of bed)**	**0.001**	**0.514**	**0.0%**	656.9	22	< 0.0001	0.3	0.064
	**Gender**	**(N. Male/Total sample)**	**0.48**	**<0.001**	**41.7%**	498.6	28	<0.0001	0.2	0.149
	Age	(Mean Age)	-0.01	0.164	3.3%	680.5	27	<0.0001	0.2	0.081
	Length of Stay	(Average days)	-0.003	0.928	0.0%	207.5	13	<0.001	0.3	0.202
	Admission	(N. adm/Total sample)	0.05	0.081	3.9%	1037.1	32	<0.0001	0.2	0.139
	**Involuntary admission**	**(N. inv-adm/Total sample)**	**0.11**	**0.003**	**26.5%**	493. 6	20	<0.0001	0.3	0.062
	Bipolar disorder	(N. Bipolar/Total sample)	-0.06	0.739	0.0%	744.2	18	<0.0001	0.3	0.074
	**Schizophrenia disorder**	**(N. schiz./Total sample)**	**0.26**	**0.005**	**20.5%**	708.9	26	<0.0001	0.2	0.129
	Personality disorder	(N. pers.dis./Total sample)	0.40	0.083	14.0%	522.3	16	<0.0001	0.3	0.151
	**Alcohol abuse**	**(N.alc.abuse/Total sample)**	**0.32**	**0.001**	**35.3%**	547.6	19	<0.0001	0.2	0.125
	**History of violence**	**(N. pts with history of violence/Total sample)**	**0.27**	**0.051**	**39.7%**	22.5	5	0.0004	0.6	0.069
**Multivariable Model 1**					68.3%	133.6	10	<0.001	0.2	0.240
Variables of the multivariable Model 1	**Gender**	**(N. Male/Total sample)**	**0.28**	**0.048**	-	-	-	-	-	-
	Involuntary admission	(N. inv-adm/Total sample)	0.06	0.151	-	-	-	-	-	-
	Schizophrenia disorder	(N. schiz./Total sample)	0.16	0.316	-	-	-	-	-	-
	**Alcohol abuse**	**(N.alc.abuse/Total sample)**	**0.21**	**0.013**	-	-	-	-	-	-
**Multivariable Model 2**					100%	0.03	1	0.855	0.6	0.136
Variables of the multivariable Model 2	Gender	(N. Male/Total sample)	-0.21	0.109	-	-	-	-	-	-
	**History of violence**	**(N. pts with history of violence/Total sample)**	**0.42**	**<0.0001**	-	-	-	-	-	-
	Schizophrenia disorder	(N. schiz./Total sample)	-0.05	0.713	-	-	-	-	-	-
	Alcohol abuse	(N.alc.abuse/Total sample)	-0.07	0.640	-	-	-	-	-	-

R^2^: regression goodness of fit index: % of explained (by covariate) heterogeneity on total heterogeneity; Q_E_: Q statistic for residual (after considering covariates) heterogeneity.

A initial multivariable regression model that included all the moderator variables that were significantly related to violence prevalence, apart from a history of violence, found that gender, involuntary admission, a diagnosis of schizophrenia and alcohol abuse explained 68% of the study heterogeneity. Information about lifetime history of violence was present only in seven studies which prevented us from including this variable in a comprehensive multivariable model. A second multivariable model, including history of violence instead of involuntary admission, was able to explain the total (100%) of the study heterogeneity (QE = 0.03, df = 1, p = 0.855). The impact of the history of violence in explaining heterogeneity was confirmed also through two bivariable meta-regression models: 97.5% of the heterogeneity was explained by history of violence together with gender; 93.8% of the heterogeneity was explained by history of violence together with diagnosis of schizophrenia.

Begg’s rank correlation test and the “Trim and fill” procedure showed substantially no publication bias. Begg’s test resulted significant only for the subgroup relating to year of publication moderator (1995–2004 category).

## Discussion

The main finding was that that almost 1 in 5 patients admitted to acute psychiatric wards in high-income countries commit an act of physical violence while in hospital. While this figure might be an important benchmark for psychiatric services, the high level of heterogeneity in the rates of inpatient violence indicates that the result does not apply to all acute inpatient settings. Wards with higher proportions of males, involuntary patients and patients with alcohol use disorders had higher proportions of patients who committed acts of violence. This finding is consistent with findings from studies on the associations with violence at individual patient level. For example, male sex and substance use disorders have been found to be reliably associated with aggression both in individual studies [28–30] and in meta-analysis [15]. Both male sex and alcohol abuse are strongly associated with violence among mentally ill outpatients, and by non-mentally disordered people in the general community.

### Gender and involuntary hospitalization

The extent to which the proportion of male patients explained the proportion of patients in an acute ward who committed an act of physical violence was somewhat surprising. Although being male is strongly associated with violence in the community, Dack and colleagues [15] found that the male sex was not strongly associated with violence at an individual patient level. Our finding suggests that the proportion of males might exert a stronger effect on the proportion of patients involved in violent confrontations though more complex interpersonal processes that operate in predominantly male populations, or where the number of females is small.

Our finding that the proportion of involuntary patients admitted under provisions of mental health laws is associated with the overall proportion of inpatient violence is also consistent with the findings of patient level data [15]. However, the association between involuntary admission and violence is also likely to be complex. First, evidence that a person is a danger to themselves or to others is a requirement for involuntary admission in many jurisdictions, creating a high threshold for treatment and, in effect, selecting patients who have been violent or who appear very likely to commit an act of violence [31]. Moreover, the process of involuntary admission and detention in a locked ward can amplify the patient’s hostility and propensity to violence, especially if they do not recognize the need for treatment.

The results of this meta-analysis of ward factors differ from those of individual patient factors in that we found that younger mean age and the proportion of patients with a diagnosis of schizophrenia did not appear to be independently associated with an increased proportion of violent patients. This might be because of the strength of the associations between these factors and violence by individual patients was small. For example, in the recent meta-analysis by Dack et al [15] the overall effect size of age (0.32 standardised mean difference) and schizophrenia (relative ratio = 1.16) was weak. In this respect, the factors associated with violence among inpatients differ from those found in studies of violence in community settings, where the presence of psychotic illness and young age is quite strongly associated with violence.

### Alcohol and substance abuse

Another factor we found to be associated to a major risk to physical violence is a history of alcohol use disorder. Our results are consistent with a previous study on acute wards in the United Kingdom, which reported no association between drug use and violence, but did find a relationship for alcohol use [32].

We also gathered the variable history of drug use or abuse, but we could not include it into the analyses, because most studies did not report these data. There is a well-established link between substance use and violence in the severely mentally ill, although the nature of the relationship is complex. Substance use can increase the risk of violence, but this may be mediated by psychiatric symptoms and social factors [33]. A systematic review concluded that while schizophrenia and psychoses can be considered general risk factors for violence, substance abuse increases the risk regardless of whether or not this is accompanied by a comorbid diagnosis [34]. Only few empirical studies have examined the links between substance use and violence in psychiatric wards [35–37] and the results of these studies suggested that substance use was a contributing factor in only 2% of cases.

### History of violence

An important predictor of violence, including while in hospital, is the lifetime history of violence. The contribution of this factor to the prevalence of violence was confirmed by the results of both univariate and multivariable meta-regression models. This finding is in line with studies [38–42] which demonstrate that a history of violence together with male gender are the most important predictors of future violence in inpatients as well as outpatients. Our findings about history of violence appeared inconsistent with those of other studies as we found a high proportion of explained variability together with high residual heterogeneity in univariate meta-regression model, as well as unstable coefficient estimates (with negative signs) in multivariable models. This is due both to the small number of studies that reported this variable (7/35), and to high correlation between proportion of patients with history of violence and proportion of male patients (Spearman correlation rho = 0.77). More information about the lifetime history of violence might have allowed a more consistent interpretation and generalization of the results concerning the relationship between this factor and the likelihood of violence while in hospital.

We did not find a higher proportion of violent patients in units with a longer average length of stay, which again differs from the results of studies examining factors associated with violence in individual patients. At an individual patient level, violence could be the reason for a longer admission, and a longer period at risk might also be associated with a higher probability of becoming involved in a violent altercation. However, at an aggregate level, wards with shorter average length of stay would be expected to have a higher turnover of acute patients than wards with a longer length of stay, and the days immediately after admission have been associated with the most serious forms of inpatient violence [43].

The possibility of a publication bias associated with the year of publication was raised by the significant Begg’s test.

However, the finding could also be due to improvements in research methodology, and improvements in the management of violent behavior, and also the declining rates of violence in the wider communities of many high-income countries during the 20 years of the study.

### Limitations of the study

First, we did not include unpublished studies, dissertations or abstract from conference proceedings, in order to limit the meta-analysis to higher quality, peer reviewed studies. We attempted to address this limitation, by examining the heterogeneity of findings and the possibility of publication bias.

Another limitation to our findings stemmed from the very high degree of between study heterogeneity in the proportion of violent patients, and the possibility of ecological fallacy in the associations between aggregate study level characteristics and the proportion of violent subjects. The between study heterogeneity among the 35 published studies is so great that there is no proportion of violent subjects that can be described as typical for an individual inpatient setting. This suggests that the results of this meta-analysis should be interpreted with caution, and that comparing rates of violence with an estimate derived from a range of high-income countries or even with other units cannot be used as a benchmark for the standard of care, and the only reasonable approach would be to consider changes in rates of violence in individual units over time.

A further limitation relates to the interpretation of the factors that we found to be associated with a higher proportion of violent incidents. For example, although it is known that male patients are more likely to be violent than female patients, the high rates of violence in the wards with more male patients might also be due to females being more violent in predominantly male wards, or because males are more violent in predominantly male wards. The same may be true for other factors such as the proportion of involuntary patients or patients with a history of alcohol or alcohol abuse: we were unable to examine whether the rates of violence in wards with larger numbers of patients with these characteristics was a result of violence by involuntary patients or patients with alcohol abuse.

A final limitation of our study is that we were not able to examine some of the factors that are associated with violence by patients at a ward level. For example, although a history of violence is one of the strongest predictors of future violence, there was insufficient data to examine the proportions of patients with a history of violence, which might also have allowed us to draw some inferences about the level of violence in the communities in which the wards were located.

There was also a lack of information about the characteristics of the wards that might influence rates of violence. Psychiatric wards differ greatly in their design, staffing and ward rules. Factors such as levels of surveillance, visibility, ward door-locking policies, ward size and spaciousness might all be important, as might the degree of tolerance of aggression, and the use of preventative strategies such as de-escalation, as required medication, time out and even seclusion. The extent of social support both within the ward with staff and patients, and outside the ward with family and friends is also likely to be important [15]. We were unable to examine the characteristics of the individual wards to identify the degree of heterogeneity that was due to potentially modifiable factors in the wards themselves.

## Conclusion

Rates of violence in acute psychiatric wards in all high-income countries appear to be both disturbingly high and highly variable between wards and settings. In general, settings with larger numbers of patients who have risk factors for violence appear to report higher levels of violence. While this result reinforces the importance of the common risk factors of male gender and alcohol use, it remains highly possible that modifiable factors in the wards themselves play an important role in determining rates of inpatient violence. Measuring rates of violence, and understanding the factors associated with individual episodes of violence are an important part of devising strategies to protect patients and staff from violent acts.

## Implications and Future Research

Establishing the risk factors for violence in acute psychiatric inpatients may enable researchers and clinicians to devise strategies to prevent and manage violence in psychiatric wards. This study confirms that important considerations include the patients’ age, diagnosis, degree of psychiatric impairment, previous history of violence, style of relating to others and response to being detained in hospital as an involuntary patient. Future research might consider the importance of any history of past violence, including violence at the time of admission to hospital, the appropriateness of the ward design for managing violent patients, staffing levels and the level of violence in the catchment area from which the patients were drawn.

## Supporting Information

S1 PRISMA Checklist PRISMA Checklist. (PDF)

S1 Fig Flow diagram of the study selection process (According to PRISMA, 2009). (PDF)
