# Supplementary material for: Prevalence and Risk Factors of Violence by Psychiatric Acute Inpatients: A Systematic Review and Meta-Analysis
Source: PLoS One. 2015 Jun 10;10(6):e0128536. doi: 10.1371/journal.pone.0128536 (PMC4464653; doi:10.1371/journal.pone.0128536)
Supplement: S1 Fig — (PDF) [file pone.0128536.s002.pdf]

PRISMA 2009 Flow Diagram

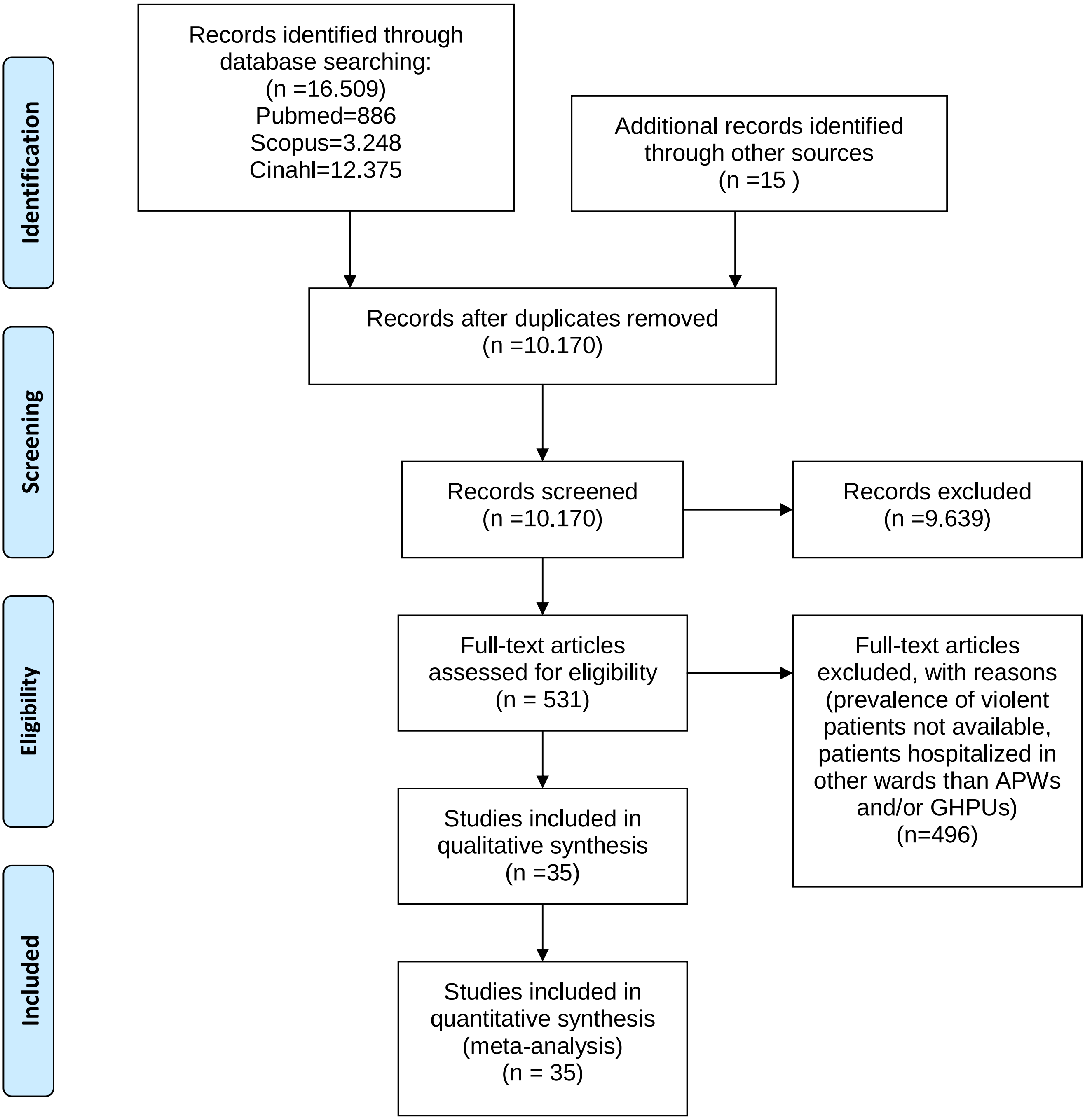

From: Moher D, Liberati A, Tetzlaff J, Altman DG, The PRISMA Group (2009). *P*referred *R*eporting *I*tems for *S*ystematic Reviews and *M*eta-*A*nalyses: The PRISMA Statement. PLoS Med 6(6): e1000097. doi:10.1371/journal.pmed1000097

For more information, visit www.prisma-statement.org.
